# Further Spread of *bla*_NDM-5_ in *Enterobacteriaceae* via IncX3 Plasmids in Shanghai, China

**DOI:** 10.3389/fmicb.2016.00424

**Published:** 2016-03-30

**Authors:** Fangfang Zhang, Lianyan Xie, Xiaoli Wang, Lizhong Han, Xiaokui Guo, Yuxing Ni, Hongping Qu, Jingyong Sun

**Affiliations:** ^1^Department of Clinical Microbiology, Ruijin Hospital, Shanghai Jiaotong University School of MedicineShanghai, China; ^2^Department of Critical Care Medicine and Respiratory Intensive Care Unit, Ruijin Hospital, Shanghai Jiaotong University School of MedicineShanghai, China; ^3^Department of Medical Microbiology and Parasitology, Institutes of Medical Sciences, Shanghai Jiaotong University School of MedicineShanghai, China

**Keywords:** *Enterobacteriaceae*, carbapenemase, NDM-5, IncX3 type plasmid, epidemiology

## Abstract

One hundred and two carbapenem-resistant *Enterobacteriaceae* (CRE) strains were isolated in a teaching hospital in Shanghai, China from 2012 to 2015. In a follow-up study, four New Delhi metallo-β-lactamase-5 (NDM-5)-producing strains were identified after screening these CRE strains, including 1 *Klebsiella pneumoniae* strain (RJ01), 1 *Proteus mirabilis* strain (RJ02), and 2 *Escherichia coli* strains (RJ03 and RJ04). All *K. pneumoniae* and *E. coli* isolates were resistant to carbapenems, third-generation cephalosporins, and piperacillin-tazobactam, but were susceptible to amikacin. No epidemiological links for either *E. coli* isolate were found by multilocus sequence typing (MLST) and pulsed-field gel electrophoresis (PFGE). However, MLST revealed a novel sequence type, ST2250, of the *K. pneumoniae* RJ01 strain. Inc types and sizes of *bla*_NDM-5_-carrying plasmids differed among the four isolates, although in *P. mirabilis* RJ02 and *E. coli* RJ03, *bla*_NDM-5_ was carried by conjugative IncX3 plasmids of nearly the same size (∼40 kb). Investigation of the genetic background of sequences flanking the *bla*_NDM-5_ gene showed that all four isolates shared the same genetic content (IS*3000*-ΔIS*Aba125*-IS*5*-*bla*_NDM-5_-*ble*-*trpF*-*dsbC*-IS*26*-Δ*umuD*), which was identical to that of the pNDM_MGR194 plasmid circulating in India. This is the first identification of *bla*_NDM-5_ in *P. mirabilis*, which suggests its further spread to *Enterobacteriaceae*, and indicates that IncX3 plasmids may play an important role in potentiating the spread of *bla*_NDM_.

## Introduction

Carbapenemase-producing *Enterobacteriaceae* have become a challenge to clinical therapy because of the rapid worldwide dissemination of multi-drug resistance (Tangden and Giske, 2015). Among the newly emerged carbapenemases, New Delhi metallo-β-lactamase-1 (NDM-1)-producing strains, which are capable of hydrolyzing all β-lactams, but not monobactams, show high potential to cause a global health crisis (Moellering, 2010). In contrast to NDM-1, NDM-2 shows low affinity for penicillin, which implies that penicillin is perhaps a better option for treating *Acinetobacter baumannii* harboring NDM-2 (Tiwari and Moganty, 2013).

New Delhi metallo-β-lactamase-5, produced by an *Escherichia coli* strain, was first identified in the UK in 2011 from a patient with a recent history of hospitalization in India (Hornsey et al., 2011). The NDM-5 enzyme differed from NDM-1 by only two amino acid substitutions (Val88Leu and Met154Leu) and showed increased resistance to carbapenems and broad-spectrum cephalosporins. Since then, NDM-5-producing strains have also been identified in Algeria (Sassi et al., 2014), China (Yang et al., 2014), Japan (Nakano et al., 2014), Spain (Pitart et al., 2015), India (Krishnaraju et al., 2015), the United States (de Man et al., 2015), South Korea (Cho et al., 2015), and Australia (Wailan et al., 2015). In 2015, clones related to NDM-5-producing strains were reported in Denmark (five isolates) (Hammerum et al., 2015) and the Netherlands (Bathoorn et al., 2015). The widespread occurrence of NDM-5 in recent years highlights the need for international attention.

To date, the gene encoding NDM-5, *bla*_NDM-5_, has only been reported in *E. coli* and *Klebsiella pneumoniae*. To evaluate the potential transmission of *bla*_NDM-5_-harboring bacterial strains in Shanghai, we screened for NDM-5-producing *Enterobacteriaceae*, and *bla*_NDM-5_ was amplified from four isolates by PCR for a more comprehensive study of the gene.

## Materials and Methods

### Bacterial Strains, Detection of Carbapenemase-encoding Genes, and Antimicrobial Susceptibility Testing

From 2012 to 2015, 102 carbapenem-resistant *Enterobacteriaceae* (CRE) strains were isolated using the VITEK^®^ two Compact system (bioMérieux, Durham, NC, USA) in the clinical microbiology laboratory of Ruijin Hospital in Shanghai, China. In a retrospective study, we amplified common carbapenemase genes (*bla*_KPC_, *bla*_IMP_, *bla*_V IM_, *bla*_OXA-48_, and *bla*_NDM_) (Nordmann et al., 2011) from all 102 CRE isolates and sequenced the positive products; four *bla*_NDM-5_-carrying strains were identified for further study. Among them, two strains were isolated in January 2014, and the other two strains were isolated in July and September of the same year, respectively. The minimum inhibitory concentrations (MICs) of amikacin, ciprofloxacin, ceftazidime, ceftriaxone, meropenem, imipenem, ertapenem, cefepime, piperacillin-tazobactam, and aztreonam were determined by the *E*-test (bioMérieux, France), according to the Clinical and Laboratory Standards Institute (CLSI) guidelines (M100-S25) (Clinical and Laboratory Standards Institute [CLSI], 2015). *E. coli* ATCC25922 was used for quality control.

### Determination of Genetic Relatedness

Multilocus sequence typing (MLST) was performed on the *E. coli*^1^ and *K. pneumoniae*^2^ isolates, respectively. Two *E. coli* isolates were further analyzed by pulsed-field gel electrophoresis (PFGE). *Salmonella enterica* serotype Braenderup H9812 was used as a size marker. Restriction patterns were compared visually and interpreted on the basis of previously defined criteria (Tenover et al., 1995).

### Plasmid Conjugation and Incompatibility Typing

A plasmid-conjugation experiment was performed between the four *bla*_NDM-5_-positive isolates and sodium azide-resistant *E. coli* J53Az^R^ as the recipient strain. Transconjugants were selected on MacConkey agar plates containing 125 mg/L sodium azide and 1.0 mg/L imipenem. Antimicrobial susceptibility testing and PCR amplification of the transconjugants were subsequently performed to confirm whether the plasmid was successfully transferred to the recipient. Plasmid incompatibility types of the isolates were identified by PCR-based replicon typing (Carattoli et al., 2005). The positive products were sequenced and used to make digoxigenin (DIG)-labeled specific probes to identify the *bla*_NDM-5_-carring plasmid.

### S1-PFGE and Southern Blotting

Plasmid-harboring bacteria were digested with S1 nuclease after being embedded in 1% SeaKem Gold Agarose gels (Lonza, Rockland, ME, USA). PFGE was performed as described above. The plasmid DNA was then transferred to positively charged nylon membranes (Roche Diagnostics GmbH Mannheim, Germany) and hybridized against DIG-labeled *bla*_NDM-5_-specific probes. Incompatibility group identification for *bla*_NDM-5_-carrying plasmids was conducted by hybridizing against *bla*_IncX3_ and *bla*_IncFII_-specific probes in the same manner.

### Analysis of the Genetic Background Flanking the *bla*_NDM-5_ Gene

Primers were designed based on the reported *bla*_NDM-5_-flanking sequences to determine the genetic background of the *bla*_NDM-5_-harboring strains (Supplementary Table S1). PCR experiments were performed using the same thermocycling conditions for all strains, as follows: one cycle of 94°C for 4 min; followed by 35 cycles of 94°C for 30 s, 58°C for 40 s, and 72°C for 1 min; and a final cycle at 72°C for 10 min. The amplification products were sequenced and compared with sequences deposited in the BLAST database^3^.

## Results

### Bacterial Strains and Antimicrobial Susceptibility Testing

In this study, four *bla*_NDM-5_-positive isolates (RJ01–RJ04) were recovered from three hospitalized patients and one outpatient in Ruijin Hospital. One hospitalized patient with *bla*_NDM-5_-carrying *E. coli* isolated from a rectal swab was found to be a carrier. In contrast, the other three patients from whom *bla*_NDM-5_-carrying strains were isolated from drainage fluid, a wound site, or mid-stream urine were symptomatic. Other patients in the same ward were also screened, but no CRE isolates were observed. None of the patients had ever been abroad. Among the isolates, RJ01 was a *K. pneumoniae* isolate, RJ02 was a *Proteus mirabilis* isolate, and RJ03 and RJ04 were *E. coli* isolates. All *E. coli* and *K. pneumoniae* isolates were resistant to carbapenems, third-generation cephalosporins, and piperacillin-tazobactam, but were susceptible to amikacin (**Table 1**). Notably, RJ04 was resistant to aztreonam, suggesting the coexistence of other resistance mechanisms. *P. mirabilis* RJ02 was highly resistant to imipenem, but was susceptible and showed intermediate resistance to meropenem and ertapenem, respectively.

**Table 1:** Antimicrobial susceptibility of the four NDM-5-producing isolates and their transconjugants.

	Plasmids carrying *bla*_NDM-5_		MIC (μg/mL)^b^										
Strain^a^	Replicon typing	Size (kb)	AMK	CIP	CAZ	CRO	MEM	IPM	ETP	FEP	TZP	ATM	Genetic environment around *bla*_NDM-5_
*Klebsiella pneumoniae* RJ01	FII	∼30	2	0.047	>256	>256	>32	>32	>32	12	>256	0.032	IS*3000*-ΔIS*Aba125*-IS*5*-*bla*_NDM-5_-*ble*-*trpF*-*dsbC*-IS*26*-Δ*umuD*
*Proteus mirabilis* RJ02	IncX3	∼40	3	>32	>256	32	1	>32	1	12	24	0.16	IS*3000*-ΔIS*Aba125*-IS*5*-*bla*_NDM-5_-*ble*-*trpF*-*dsbC*-IS*26*-Δ*umuD*
Tc-RJ02	IncX3	∼40	1	0.023	>256	>256	4	>32	8	12	>256	0.47	IS*3000*-ΔIS*Aba125*-IS*5*-*bla*_NDM-5_-*ble*-*trpF*-*dsbC*-IS*26*-Δ*umuD*
*Escherichia coli* RJ03	IncX3	∼40	4	>32	>256	>256	>32	>32	>32	48	>256	0.47	IS*3000*-ΔIS*Aba125*-IS*5*-*bla*_NDM-5_-*ble*-*trpF*-*dsbC*-IS*26*-Δ*umuD*
Tc-RJ03	IncX3	∼40	1	0.012	>256	>256	>32	>32	>32	16	>256	0.47	IS*3000*-ΔIS*Aba125*-IS*5*-*bla*_NDM-5_-*ble*-*trpF*-*dsbC*-IS*26*-Δ*umuD*
*E. coli* RJ04	IncX3	∼180	3	>32	>256	>256	>32	>32	>32	>256	>256	>256	IS*3000*-ΔIS*Aba125*-IS*5*-*bla*_NDM-5_-*ble*-*trpF*-*dsbC*-IS*26*-Δ*umuD*

*^a^Tc-, transconjugant strain. ^b^Antibiotics: AMK, amikacin; CIP, ciprofloxacin; CAZ, ceftazidime; CRO, ceftriaxone; MEM, meropenem; IPM, imipenem; ETP, ertapenem; FEP, cefepime; TZP, piperacillin-tazobactam; ATM, aztreonam.*

### Genetic Relatedness of Four Isolates

According to the MLST results, *E. coli* RJ03 and *E. coli* RJ04 belonged to sequence type (ST) 354 and ST156, respectively. In accordance with the MLST results, the differences of the PFGE patterns confirmed that the two *E. coli* isolates are not clonally related. Because the sequence of *K. pneumoniae* RJ01 did not match any known STs, it was assigned a novel sequence type, ST2250 (10-20-2-1-9-11-25).

### Characteristics of the *bla*_NDM-5_-Carrying Plasmids

**FIGURE 1:**
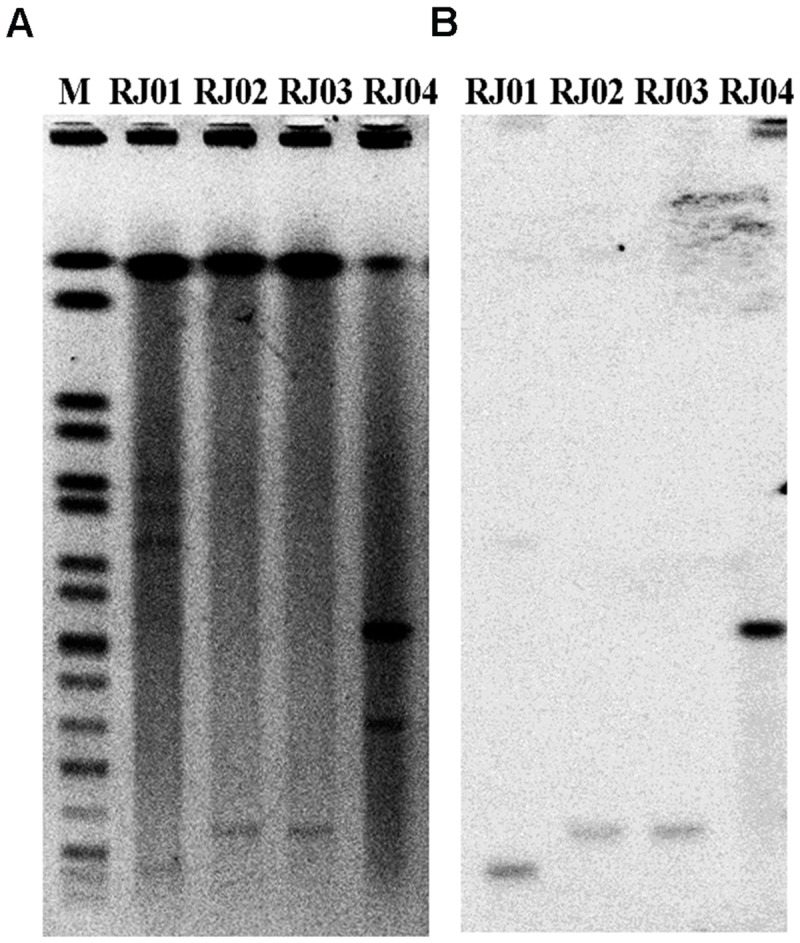
**S1-pulsed-field gel electrophoresis (S1-PFGE) patterns of the four *bla*_NDM-5_-producing isolates (A) and Southern hybridization with a probe specific for *bla*_NDM-5_ (B).** M, marker; *Salmonella enterica* serotype Braenderup H9812.

In our study, RJ02 and RJ03 successfully transferred *bla*_NDM-5_ to the recipient, whereas RJ01 and RJ04 did not. An antimicrobial susceptibility test and PCR amplification were performed to confirm the presence of the gene and its phenotype in the transconjugants. S1-PFGE and subsequent Southern hybridization against DIG-labeled *bla*_NDM-5_ with incompatibility group-specific probes revealed that *bla*_NDM-5_ was carried by IncX3 plasmids of nearly the same size (∼40 kb) in *P. mirabilis* RJ02 and *E. coli* RJ03 (**Figure 1**), by a ∼180-kb IncX3 plasmid in the case of *E. coli* RJ04, and by a ∼30-kb IncFII plasmid in the case of *K. pneumoniae* RJ01.

### Genetic Surroundings of *bla*_NDM-5_

All four *bla*_NDM_-_5_-positive isolates were found to have an identical genetic background, IS*3000*-ΔIS*Aba125*-IS*5*-*bla*_NDM-5_-*ble*-*trpF*-*dsbC*-IS*26*-Δ*umuD*, which is the same as that of isolate pNDM_MGR194 in India (GenBank Accession Number KF220657) (Krishnaraju et al., 2015).

## Discussion

Since *bla*_NDM_*_-5_* was first identified in the UK (Hornsey et al., 2011), strains harboring this gene have emerged in several countries (Sassi et al., 2014; Yang et al., 2014; de Man et al., 2015; Krishnaraju et al., 2015; Pitart et al., 2015; Wailan et al., 2015). Because NDM-5 has only been previously reported in *E. coli* and *K. pneumoniae*, the NDM-5 found in *P. mirabilis* RJ02 indicates the further spread of *bla*_NDM-5_ among different species of *Enterobacteriaceae.* Although NDM-5-producing strains are not as widespread as NDM-1-producing strains, their greater resistance to antimicrobial drugs make them a potential public health threat.

*E. coli* ST354 and ST156 were found in our study. Although ST648 has been reported both in the UK (Hornsey et al., 2011) and in Australia (Wailan et al., 2015), most reports have indicated a high ST diversity for *bla*_NDM-5_-positive *E. coli* (Sassi et al., 2014; Yang et al., 2014; Cho et al., 2015; Pitart et al., 2015). From 2014 to 2015, six NDM-5-producing ST16 *K. pneumoniae* isolates were identified among patients in Denmark (five isolates) (Hammerum et al., 2015) and the Netherlands (one isolate) (Bathoorn et al., 2015), who had not traveled recently. Before these reports were published, no other outbreaks of NDM-5-producing Gram-negative bacteria were reported. Although no evident genetic association was found between our *bla*_NDM-5_-positive isolates (ST2250) with other strains, the first report of an NDM-5-related outbreak in Europe showed an epidemic potential and deserved extensive attention.

A previous study conducted in Algeria showed that all three *bla*_NDM-5_-positive *E. coli* strains tested could transfer their resistant plasmids to the azide-resistant *E. coli* strain J53 (Sassi et al., 2014), which implied the possibility of horizontal transfer of the *bla*_NDM-5_ gene. In our study, two strains successfully transferred *bla*_NDM-5_ to the recipient, thereby reconfirming the horizontal transfer ability. IncX3 plasmids might have played an important role in mediating the horizontal transmission of *bla*_NDM_ (Ho et al., 2012; Sonnevend et al., 2013), a possibility that has been supported by the results of several studies (Gottig et al., 2013; Yang et al., 2014; Krishnaraju et al., 2015). Here, *bla*_NDM-5_ was carried by IncX3 plasmids in three strains in our study. To date, IncX3 plasmids carrying *bla*_NDM-5_ have been reported in China (Yang et al., 2014), India (Krishnaraju et al., 2015), Australia (Wailan et al., 2015), and Denmark (Hammerum et al., 2015). Therefore, our current study complements these previous data. IncX-type plasmids have a narrow host range in *Enterobacteriaceae.* The fact that IncX-type plasmids have been shown to be conjugatable in most studies could explain the rapid spread of NDM-carrying isolates. Therefore, it is imperative that effective and feasible measures are taken immediately to control the dissemination of these resistant plasmids.

India is considered to be one the main reservoirs of *bla*_NDM_ (Nordmann, 2014). Travel to endemic countries is generally associated with the global dissemination of carbapenemase-producing *Enterobacteriaceae* (Savard and Perl, 2014). Although the four isolates shared the same genetic background with isolate pNDM_MGR194 from India (GenBank Accession Number KF220657) (Krishnaraju et al., 2015), none of the four patients had ever been abroad. Previous reports of *bla*_NDM_-containing plasmids isolated from patients in China also showed little contact with the Indian subcontinent (Wang et al., 2012). However, as international travel is becoming increasingly common, some routes of transmission between people might be unrecognized. We further compared the genetic environments flanking the *bla*_NDM-5_ genes among isolates from China (Yang et al., 2014), India (Krishnaraju et al., 2015), and Japan (Nakano et al., 2014) and found that they were nearly identical, except for the number and position of the IS*5* insertion. Therefore, we speculate that horizontal transference has played an important role in dissemination, although more data are needed to explain how the *bla*_NDM-5_ gene has spread among these countries.

In summary, our study provides evidence for the further spread of the *bla*_NDM-5_ gene in Enterobacteriaceae. These results thus expand previous data on NDM-5-producing strains, and support the speculation that the IncX3-type plasmids have played a major role in the global dissemination of NDM-producing *Enterobacteriaceae*. This work strongly highlights the urgent need for effective action to control the horizontal spread of *bla*_NDM-5_.

## Author Contributions

FZ, LX, XW, LH, XG, YN, HQ, and JS: substantial contributions to the conception and design of the work; revising it critically for important intellectual content; final approval of the version to be published; agreement to be accountable for all aspects of the work in ensuring that questions related to the accuracy or integrity of any part of the work are appropriately investigated and resolved; analyzed the data; contributed materials.

## Conflict of Interest Statement

The authors declare that the research was conducted in the absence of any commercial or financial relationships that could be construed as a potential conflict of interest.

## Footnotes

1 http://mlst.warwick.ac.uk/mlst/dbs/Ecoli

2 http://www.pasteur.fr/recherche/genopole/PF8/mlst/Kpneumoniae.html

3 http://www.ncbi.nlm.nih.gov/BLAST
