# Supplementary material for: Further Spread of blaNDM-5 in Enterobacteriaceae via IncX3 Plasmids in Shanghai, China
Source: Front Microbiol. 2016 Mar 30;7:424. doi: 10.3389/fmicb.2016.00424 (PMC4811927; doi:10.3389/fmicb.2016.00424)
Supplement: Supplementary file 1 [file Table_1.DOC]

**Supplementary Table 1. Sequences of primers designed for this study.**

| **Primer** | **Sequence(5'–3')** | **Product length(bp)** |
| --- | --- | --- |
| **For5198** | GCTCATAGTAGATTTTGGGGG | 1065 |
| **Re6262** | GGAGTTCGTAGGGATGCAGTT |
| **For6154** | TGCAGCTCCATCAGCAAAAGG | 1177 |
| **Re7330** | TCGGGTGAAGTCGGGAAAATC |
| **For6978** | GCCCCTATTCTCTCGGCTTTC | 1285 |
| **Re8262** | TTCTTCCCCTATCCTGACCTC |
| **For7924** | GGCGACGCTGGATAGAACA | 939 |
| **Re8862** | ACTCACGCGCATCAGGACA |
| **For8568** | ACTTGGCCTTGCTGTCCTT | 1363 |
| **Re9930** | GCGTTGCTGCTCTTTGTTC |
| **For9514** | GAGATTTTCTTGTCCCGCA | 1081 |
| **Re10594** | TTGGCTTACACCATTAGGG |
| **For10318** | AAAGCCAGATACAAGGGGT | 935 |
| **Re11252** | CTCAGCAAATAGCAAAGCG |
| **For11016** | TTTCTTGAACTTCGGCTGG | 1001 |
| **Re12016** | GCTATGGAATGAGTGCGCT |
| **For11882** | GGGGATGCCTGTTTGACGA | 1147 |
| **Re13028** | CGGGGATTGCGGATGTACT |
| **For12660** | AACGGGTTTTGAAGGTCTC | 1169 |
| **Re13828** | AGCGGTGTTTAAGGATTGG |
| **For13486** | GCCGTTGAGTTCGTTTTGT | 1343 |
| **Re14828** | CCCTATTTGGGCTCTGTTT |
| **NDM-F** | GGTTTGGCGATCTGGTTTTC | 621^11^ |
| **NDM-R** | CGGAATGGCTCATCACGATC |
